# An Underestimation Bias in the Numerical Perception of Rewarding Stimuli: An ERP Study

**DOI:** 10.3390/jintelligence14030043

**Published:** 2026-03-05

**Authors:** Xingyuan Xue, Yuan Yao

**Affiliations:** Department of Psychology, Suzhou University of Science and Technology, No. 99 Xuefu Road, Suzhou 215009, China; 13607324541@163.com

**Keywords:** number sense, reward, C1, N1, P2p

## Abstract

Number sense, the ability to rapidly perceive, estimate, and understand relationships between quantities, constitutes a fundamental basis for mathematical cognition. However, the extent to which it is modulated by top-down regulatory processes remains poorly understood. Rewards inherently carry quantitative attributes of abundance and scarcity, and prospect theory further suggests that individuals tend to underestimate rewards and overestimate punishments of equal magnitude, implying that the perception of reward quantities may be systematically biased. To address this issue, the present study employed EEG to examine how reward-related properties of stimuli modulate number sense, using socially relevant reward stimuli as experimental materials. Behavioral results demonstrated that rewarding stimuli were underestimated compared to neutral and punishing stimuli, while punishing stimuli were overestimated relative to neutral stimuli. EEG analyses revealed that at number-sensitive electrodes (PO7, PO8, Oz), the C1 component was sensitive to reward properties; the N1 component at PO7 was specifically sensitive to punishment; and in the P2p time window, neutral stimuli elicited the largest amplitudes, suggesting inhibitory processing of reward-related attributes during quantity perception. Together, these findings indicate that reward-based modulation of number sense occurs unconsciously and follows a dynamic temporal profile.

## 1. Introduction

Mathematical ability represents a cornerstone of human cognition. A fundamental component underlying mathematical learning is number sense—a cognitive capacity that reflects the brain’s extraction and representation of quantitative information from visual stimuli, forming the basis for higher-level mathematical skills such as arithmetic computation (Butterworth, 2005; Halberda et al., 2009; Van Bueren et al., 2022). Similar to the role of phonological processing in reading development, number sense serves as a reliable predictor of mathematical achievement (Ansari & Karmiloff-Smith, 2002). This capacity is widely observed across ages and species, including human infants, non-human primates, and even invertebrates (Brannon, 2006; Gross et al., 2009; Nieder et al., 2002), with evidence suggesting that number sense in infancy lays the foundation for later mathematical abilities (Starr et al., 2013). Given its critical role, a robust number sense is essential for mathematical proficiency. However, numerical processing is not a closed system; its neural substrates, such as the parietal–prefrontal network, are also modulated by multiple cognitive factors (Nieder, 2025). The medial temporal lobe (MTL) further supports arithmetic working memory through static and dynamic coding mechanisms (Kutter et al., 2022). Similar to transitive inference, number sense also exhibits a distance effect (Park et al., 2015; Surabhi et al., 2022). It is therefore imperative to investigate how number sense is modulated by other cognitive and environmental influences, such as how understanding can inform and enhance instructional practices in mathematics education.

Numerosity is considered a primary visual attribute, comparable to color, orientation, and contrast, which the visual system can process automatically to extract meaning (Park et al., 2015). Furthermore, numerical perception operates largely independently of low-level visual features such as orientation and color (Allïk & Tuulmets, 1991), and is not modality-specific, being evident across visual, auditory, and tactile domains (Starkey et al., 1990). This suggests that numerosity processing may constitute a relatively independent cognitive system. EEG studies have shown that during quantity processing, numerosity elicits greater amplitude modulations over midline occipital and bilateral occipitoparietal regions compared to other visual attributes like area or density (Park et al., 2015). Numerosity discrimination begins in the early visual cortex, as indicated by the sensitivity of the C1 and P2p ERP components to numerical information (Fornaciai et al., 2017). A fully developed number sense emerges as early as the V3 area in the occipital cortex (Fornaciai & Park, 2018), and the first negative component (N1) after stimulus onset also demonstrates sensitivity to numerosity (Yao et al., 2023). Additionally, number sense shares characteristics with other sensory modalities; for instance, adaptation leads to underestimation of large numerosity and overestimation of small ones (Burr & Ross, 2008). Moreover, topological properties, such as connectivity and closure, also constrain numerical judgments, resulting in systematic underestimation (He et al., 2015), further supporting the status of numerosity as a fundamental visual attribute. In summary, evidence converges to indicate that numerosity perception is a precise process relatively independent of top-down modulation, with other factors primarily influencing it by inducing overestimation or underestimation effects. Given that adaptation and topological perception represent fundamental properties of sensory systems (Burr et al., 2025; Burr & Ross, 2008; Chen, 1982), numerosity appears to be primarily governed by such low-level perceptual mechanisms.

However, a growing body of research demonstrates that numerical perception is also susceptible to modulation by higher-order cognitive processes, including emotional states. For example, exposure to a positive emotional face prior to a numerosity judgment task has been shown to induce a systematic underestimation of quantities (Lewis et al., 2018). Moreover, individuals with high levels of math anxiety exhibit reduced precision in their Approximate Number System (ANS) compared to their non-anxious peers. Notably, even when presented with identical visual stimuli, ANS acuity is generally higher during active tasks than during passive viewing conditions (Maldonado Moscoso et al., 2020; Mielicki et al., 2024). These findings collectively suggest that top-down influences can introduce systematic biases—either overestimation or underestimation—into quantitative judgments. However, there is no empirical evidence that these biases follow predictable systematic patterns. Insights from prospect theory, particularly the principle of loss aversion, posit that the psychological impact of a loss outweighs that of an equivalent gain (Kőszegi & Rabin, 2006). This asymmetry implies that when individuals evaluate quantities associated with rewards, their judgments may not rely solely on an autonomous number sense but could be influenced by a higher-order cognitive weighting of value. Consequently, it remains unclear whether rewarding stimuli lead to systematic underestimation in number sense.

Rewards—defined as desirable, attractive, and positive outcomes of behavior—play a critical role in modulating and sustaining the frequency and intensity of associated actions (Schultz, 2015). Such motivational signals significantly influence performance across a range of visual tasks. According to value-driven attentional capture theory, stimuli previously associated with reward, even when task-irrelevant, receive prioritized attentional resources, whether the rewards are monetary or social in nature (Anderson et al., 2011). Empirical studies consistently demonstrate that both social rewards (e.g., positive facial feedback) and non-social rewards (e.g., monetary incentives) accelerate attentional allocation (Diao et al., 2024; Gao et al., 2024; Zhao et al., 2020). However, the behavioral consequences of reward are not uniform: social rewards have been shown to either enhance task performance (Gao et al., 2024; Hodsoll et al., 2011), or disrupt the identification of subsequent stimuli, thereby impairing performance (Gutiérrez-Cobo et al., 2019). Similarly, non-social reward cues can improve perceptual precision at the encoding stage (Cheng et al., 2021), and the anticipation of monetary rewards has been found to increase response accuracy (Wolf & Lappe, 2023). Neuroimaging studies suggest that reward-related modulations of attention involve dynamic interactions across multiple large-scale brain networks, including the reward circuit, default mode network, frontoparietal network, dorsal attention network, and salience network (Yankouskaya et al., 2022). These networks reconfigure their functional topology to accommodate the processing of reward and affective valence. Specifically, the medial orbitofrontal cortex represents various types of rewards, whereas the lateral orbitofrontal cortex encodes punishments (Rolls et al., 2020). Electrophysiological markers further illuminate the temporal dynamics of reward processing: the feedback-related negativity (FRN), a negative-going component peaking between 200 and 300 ms after feedback over frontal–midline sites (e.g., Fz, FCz, Cz), is typically larger following non-reward or loss outcomes compared to rewards. This component is thought to reflect dopaminergic prediction error signals originating from the anterior cingulate cortex (Glazer et al., 2018; Zhou et al., 2010). Additionally, reward motivation enhances the amplitude of the P300 component at central and parietal sites (e.g., Cz, Pz) relative to non-motivated conditions (Kaya et al., 2022). In summary, reward-induced neural changes predominantly emerge over frontal–midline and occipitoparietal regions within a time window of approximately 200–300 ms post-stimulus.

Emerging evidence suggests that social rewards can modulate number sense (Lewis et al., 2018). However, this modulation appears to stem not from the intrinsic reward properties of the numerical stimuli themselves, but rather from the carryover effects of reward-related items that are independent of the numerosity judgment task. Although extensive research has demonstrated the influence of reward on attentional processes, the majority of studies have focused on how reward-induced biases accelerate or delay reaction times in attention-oriented tasks. Few investigations have examined whether direct exposure to reward-associated stimuli elicits perceptual biases that interfere with basic perceptual processes, and the underlying cognitive and neural mechanisms of such effects remain largely unexplored.

Therefore, the present study aims to investigate whether a systematic perceptual bias occurs when humans quantify reward-related stimuli, with the specific hypothesis that individuals tend to underestimate the numerosity of reward items compared to non-rewarding items. To explore the underlying neural correlates of this effect, electroencephalography (EEG) will be employed to record brain activity during the task. In line with prior work, rewarding stimuli are categorized into social and non-social types (Gu et al., 2019; Matyjek et al., 2020; Sailer et al., 2023). Given existing evidence that happy expressions modulate number sense (Lewis et al., 2018), and to avoid overburdening participants that would result from simultaneously including non-social reward stimuli, we selected facial expression symbols from the social reward domain as experimental materials. Previous research supports the use of emojis as valid proxies for emotional expressions (Jaeger et al., 2019; Jaeger & Ares, 2017). Accordingly, we employed emoji packs as stimulus sets, with happy, neutral, and sad expressions representing the three experimental conditions. The use of emojis helps control for potential confounds such as facial gender and reduces overall participant fatigue. Indeed, although positive emotions are considered social rewards, they cannot completely decouple emotional and reward attributes—a disadvantage of facial expression stimuli given the distinct differences between the reward system and the emotional system (Chakravarthula & Padmala, 2023). Nevertheless, such stimuli offer a clear advantage; facial expressions can encompass both positive and negative valence (Russell, 1980), which represents a strength over monetary rewards, as the latter typically associate punishment with financial loss—a feature not incorporated into the reinforcement behavior within the present paradigm.

For behavioral measures, the point of subjective equality (PSE) was adopted as an indicator of numerical underestimation, consistent with previous studies (He et al., 2015). The PSE represents the physical stimulus value at which participants perceive two alternatives as equal with 50% probability (Fechner, 1948). A higher PSE reflects that a greater physical quantity is needed to support a “different” judgment, indicating perceptual underestimation; conversely, a lower PSE suggests overestimation. We hypothesized that happy stimuli would elicit a significantly higher PSE compared to neutral and sad stimuli, whereas sad stimuli would yield a significantly lower PSE relative to both neutral and happy conditions. For EEG measures, electrodes PO7, PO8, and Oz were selected as regions of interest for assessing numerosity processing, based on their established sensitivity to early visual and numerical processing (Fornaciai et al., 2017; Fornaciai & Park, 2017; Park et al., 2015; Yao et al., 2023). The C1, N1, and P2p components were chosen as target ERP markers of early visual and numerical stages. We expected that reward-related modulation would manifest in these early components, supporting the view that reward exerts an unconscious and automatic influence on number sense.

## 2. Method

### 2.1. Participant

Based on an a priori power analysis conducted using G*Power 3.1.7, a minimum sample size of 34 participants was required to achieve a statistical power of 0.8. To account for potential attrition or exclusion due to data quality issues, a total of 40 participants were recruited, ensuring adequate statistical power for the study. The experimental procedures received ethical approval from the Ethics Committee of Suzhou University of Science and Technology. All participants were right-handed, had normal or corrected-to-normal vision, reported no history of color blindness or visual deficiencies, and had no known neurological or psychiatric disorders. Prior to participation, each individual provided written informed consent and received course credit upon completion of the study. Investigations were conducted in accordance with the principles outlined in the Declaration of Helsinki (1975, revised in 2013).

### 2.2. Materials

Experimental stimulus materials were all generated using MATLAB 2022b.

Target stimuli: Different numbers of emoji symbols were drawn within a 600 × 600 pixel white (RGB: 255, 255, 255) background square. All emoji symbols were uniformly set to 70 × 70 pixels, with random positions and densities, corresponding to a visual angle of 18.32°. The number of expressions included seven levels: 9, 10, 11, 12, 13, 14, and 15. Expression types were divided into three conditions—happy faces (rewards), sad faces (punishments), and neutral faces (controls)—ultimately forming 7 (quantities) × 3 (expression types) = 21 types of target stimulus materials. Each type of stimulus was randomly generated 30 times, resulting in a total of 630 target stimuli.

**Figure 1:**
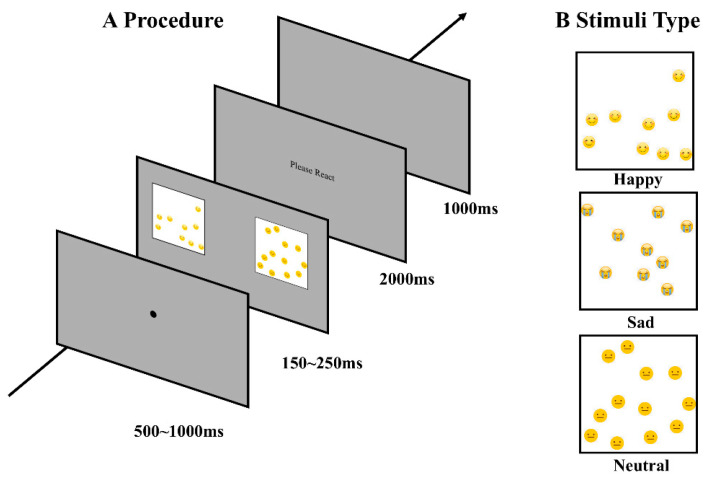
Trial Procedure and Stimuli: (**A**) Illustrates the experimental procedure; (**B**) displays all the stimuli used across the experiments.

Standard stimuli: Using the same method, 12 neutral expression symbols were fixed and used, with their positions and density distributions randomly generated. All expression symbols were 70 × 70 pixels in size, drawn within a 600 × 600 pixel white background square, generating a total of 630 standard stimuli. Material details are shown in Figure 1.

### 2.3. Procedure

The experiment was conducted in a soundproof, dark room, with participants wearing a 64-channel electrode cap, with their eyes maintaining a 60 cm distance from a 27-inch 2K resolution monitor (Chinese VOC brand DR400 model, refresh rate 144 Hz, the monitor placed on a 200 × 80 × 70 cm experimental table), corresponding to a visual angle of 18.32°. Visual stimulus presentation was controlled through the Screen function in Psychtoolbox-3 (version 3.0.18) running on the MATLAB R2022a platform. After starting the experimental program, participants first read the task instructions, then completed a practice phase to familiarize themselves with the procedure. The practice phase included only two extreme quantity conditions (9 and 15 emoji symbols) and three expression types (happy faces, sad faces, and neutral faces), with each condition repeated 3 times, for a total of 18 practice trials. Only when the participant’s accuracy rate exceeded 60% could they proceed with the formal experiment, thereby excluding random responses and providing a basis for subsequently eliminating unresponsive participants.

In the formal experiment, a gray background (RGB: 128, 128, 128) screen first presented a fixation point in the center for 500–1000 ms, followed by the standard stimulus and target stimulus presented simultaneously at balanced left and right positions on the screen (horizontally centered, eccentricity 14.2°) for 150–250 ms (presentation duration determined by frame-based time conversion: target duration divided by single frame duration and rounded, with 150 ms, 200 ms, and 250 ms conditions corresponding to 22 frames/152.78 ms, 29 frames/201.39 ms, and 36 frames/250 ms respectively). After the stimuli disappeared, a 2 s “Please respond” prompt appeared in the center of the screen. If participants believed the left dot array had more quantity, they pressed the “F” key; otherwise, they pressed the “J” key. Each trial was followed by a 1 s interval before entering the next trial. The experiment consisted of a total of 630 trials, with the entire process taking 42 min (±3 min to include individualized rest time). The procedure is shown in Figure 1.

### 2.4. Behavioral Analysis

Based on the principles of the method of constant stimuli, we established a psychophysical curve relating stimulus intensity to the frequency of “greater” judgments, and calculated the stimulus intensity at which participants produced a “greater” response probability of 50% through linear interpolation, thereby obtaining the point of subjective equality (PSE). Data analysis was performed using R-4.4.0 and RStudio 2024.04.0: first, the ‘dplyr’, ‘tidyr’, and ‘quickpsy’ packages were loaded, followed by the use of the ‘group_by’ function to calculate the “greater” response frequency for each participant under different stimulus conditions (frequency = number of “greater” judgments/total trials for that condition). The data were divided by stimulus type, and the ‘quickpsy’ function was used to fit a cumulative Gaussian model with frequency as the dependent variable and stimulus quantity as the independent variable. This model predicts the stimulus quantity corresponding to a 50% “greater” response rate, which serves as the PSE for each participant, representing the subjective equivalence point when perceiving the target stimulus as equal in quantity to the standard stimulus (fixed at 12). Finally, all participants’ PSEs were aggregated for repeated measures analysis of variance.

Statistical analysis was conducted using SPSS 27.0. Under the general linear model framework, repeated measures analysis of variance was performed: a within-subjects factor with three levels (happy, sad, neutral) was defined, and the behavioral experimental results were tested with PSE as the dependent variable.

### 2.5. Electrophysiological Recordings

EEG data were acquired using 64 Ag-AgCl electrodes arranged according to the international 10–20 system, and the signals were recorded with a sampling rate of 1000 Hz and a bandwidth of 0–80 Hz, ensuring high temporal resolution. Signal amplification was performed with the Neuroscan SynAmps2 system, which offers 24 bit resolution for precise data capture. Impedances were kept below 10 kΩ for all electrodes. All scalp electrodes and EOG signals were referenced to CPz during recording.

### 2.6. EEG Data Analysis

EEG data were preprocessed using EEGLAB. The downsampling rate was 512 Hz, bandpass filtering was performed between 0.01~30 Hz, and re-referencing was done using the whole-brain average. Subsequently, ICA analysis was conducted, rejecting “Eye” and “Muscle” artifacts with a probability range from 0.8 to 1. Bad segments were automatically removed based on a ±100 μV criterion (Keil et al., 2014). Epochs were extracted from continuously recorded EEGs relative to the onset of number pairs, 200 ms preceding and 600 ms after the stimuli. One participant was excluded because the bad segment rejection rate was too high (>20%); ultimately, 39 qualified datasets entered the final analysis. Each experimental condition had an average of 201 trials, with no significant differences in the number of trials across conditions. Using the ERPLAB plugin, the average amplitude, negative peak, and latency of the C1 (50–100 ms), N1 (170–260 ms) and P2p (270–360 ms) components at electrodes PO7, PO8, and Oz were exported. Repeated measures analysis of variance was then performed using R language to examine the main effect differences in stimulus types.

## 3. Result

### 3.1. Behavioral Results

Descriptive statistics for RT and the PSE across these conditions are detailed in Table 1.

A repeated measures ANOVA on RT across stimulus types revealed a significant main effect of stimuli type, *F*(2, 76) = 8.140, *p* < 0.001, η^2^_p_ = 0.176. Post hoc comparisons (Sidak-corrected) indicated that: the RT for the happy condition was significantly faster than the sad condition (M_diff_ = 0.013, 95% CI [0.008, 0.018], *p* = 0.012), and the neutral condition was significantly faster than the sad condition (M_diff_ = 0.017, 95% CI [0.012, 0.021], *p* = 0.001).

**Figure 2:**
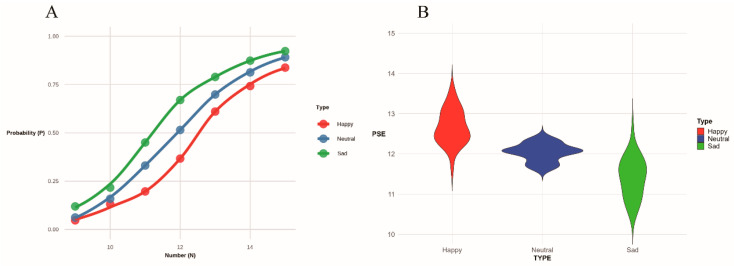
Main effect of stimulus type: (**A**) Probability of judging the quantity as greater than 12 plotted against the number of stimuli. (**B**) Distribution of the point of subjective equality across different stimulus types. Colors represent stimulus types: red (happy), blue (neutral), and green (sad).

To test whether reward attributes lead to numerical underestimation, a repeated measures ANOVA on PSE across stimulus types revealed a significant main effect of stimuli type, *F*(2, 76) = 79.900, *p* < 0.001, η^2^_p_ = 0.678. Post hoc comparisons (Sidak-corrected) indicated that: the PSE for the happy condition was significantly higher than both the sad condition (M_diff_ = 1.318, 95% CI [1.059, 1.576], *p* < 0.001) and the neutral condition (M_diff_ = 0.632, 95% CI [0.471, 0.792], *p* < 0.001). The PSE for the neutral condition was also significantly higher than that of the sad condition (M_diff_ = 0.686, 95% CI [0.483, 0.888], *p* < 0.001). The details are shown in Figure 2.

To provide a more comprehensive characterization of the effects of emotional valence on numerical perception, we fitted cumulative normal psychometric functions to each participant’s data in each condition using the quickpsy package in R (Linares & López-Moliner, 2016). In addition to the PSE, we extracted the slope parameter (defined as 1/σ, where σ is the scale parameter) and computed the just noticeable difference (JND = ln(3)/slope ≈ 1.0986/slope) as an index of perceptual precision.

All participant–condition combinations yielded successful convergence, with excellent model fit across the board (mean deviance = 0.213, mean log-likelihood = −3.28; all deviances < 1).

A repeated measures ANOVA on JND (with participant as a random effect) revealed no significant main effect of stimulus type, *F*(2, 112) = 0.094, *p* = 0.910, η^2^_p_ < 0.01. This indicates that perceptual sensitivity to numerical differences did not differ significantly across the happy, neutral, and sad conditions. Together with the significant effects observed on PSE, these results suggest that emotional valence primarily modulates numerical estimation bias rather than perceptual precision.

### 3.2. ERP Results

**Figure 3:**
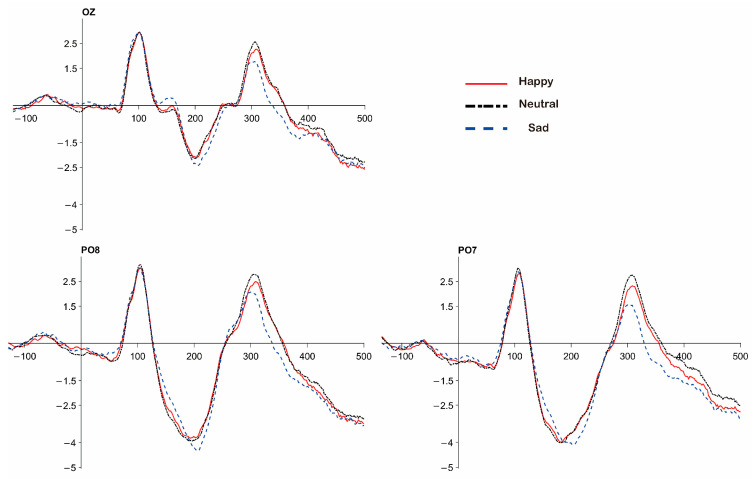
EEG from OZ, PO7, and PO8 electrodes: The red lines represent happy stimuli, the black lines represent neutral stimuli, and the blue lines represent sad stimuli. From left to right are the OZ electrode, the PO7 electrode, and the PO8 electrode.

To examine the modulation of stimulus attributes on early number sense-related brain regions, we performed ERP analysis. According to previous studies (Park et al., 2015) three electrodes (PO7, PO8 and Oz) from the parietal region were chosen. The mean amplitudes of C1 (50–100 ms), N1 (170–260 ms) and P2p (270–360 ms) in the signals recorded at the PO7, PO8 and OZ electrodes were separately analyzed. The details are shown in Figure 3. 

#### 3.2.1. Analysis of C1 Component

With stimulus type as the independent variable and mean amplitude as the dependent variable, a repeated ANOVA was performed. The results indicated that for the C1 component (50–100 ms), the main effect of stimulus type at the PO7 electrode was significant, *F*(2, 76) = 3.284, *p* = 0.043, η^2^_p_ = 0.080. Post hoc tests showed that the amplitude for happy stimuli was significantly smaller than for sad stimuli, M_diff_ = 0.224, *p* = 0.044, and the amplitude for neutral stimuli was significantly smaller than for sad stimuli, M_diff_ = 0.188, *p* = 0.040. At the PO8 electrode, the main effect of stimulus type was significant, *F*(2, 76) = 4.028, *p* = 0.022, η^2^_p_ = 0.096. Post hoc tests showed that the amplitude for happy stimuli was significantly smaller than for sad stimuli, M_diff_ = 0.182, *p* = 0.036, and the amplitude for neutral stimuli was significantly smaller than for sad stimuli, M_diff_ = 0.237, *p* = 0.018. At the Oz electrode, the main effect of stimulus type was significant, *F*(2, 76) = 4.185, *p* = 0.012, η^2^_p_ = 0.099. Post hoc tests showed that the amplitude for happy stimuli was significantly smaller than for sad stimuli, M_diff_ = 0.215, *p* = 0.010, and the amplitude for neutral stimuli was significantly smaller than for sad stimuli, M_diff_ = 0.220, *p* = 0.021.

With stimulus type as the independent variable and negative peak amplitude as the dependent variable, a repeated ANOVA was performed. The results indicated that the main effect of stimulus type at the PO8 electrode was significant, *F*(2, 76) = 6.363, *p* = 0.003, η^2^_p_ = 0.143. Post hoc tests showed that the amplitude for happy stimuli was significantly greater than for neutral stimuli, M_diff_ = 0.204, *p* = 0.031, and the amplitude for neutral stimuli was significantly smaller than for sad stimuli, M_diff_ = −0.348, *p* = 0.003.

**Figure 4:**
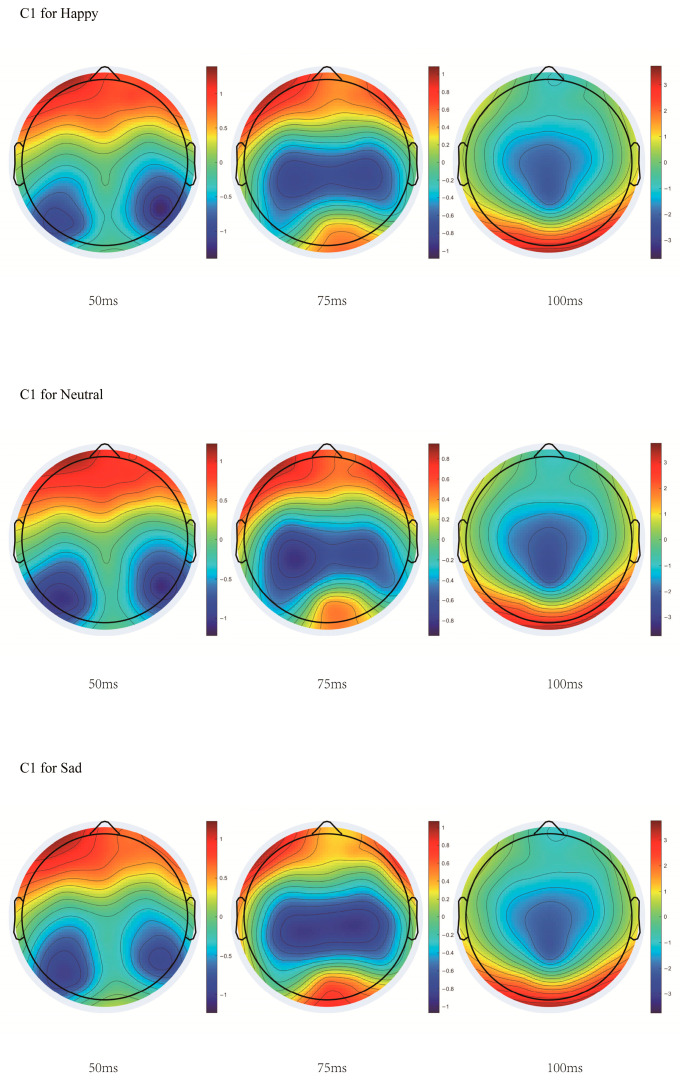
Topographic map of the C1 component.

With negative peak latency as the dependent variable and stimulus type as the independent variable, a repeated ANOVA was performed. The results indicated that the main effect of stimulus type at the OZ electrode was significant, *F*(2, 76) = 4.729, *p* = 0.012, η^2^_p_ = 0.111. Post hoc tests showed that the latency for happy stimuli was significantly shorter than for neutral stimuli, M_diff_ = 5.810, *p* = 0.022, and the latency for sad stimuli was significantly shorter than for neutral stimuli, M_diff_ = 6.510, *p* = 0.012. The details are shown in Figure 4.

#### 3.2.2. Analysis of N1 Component

With stimulus type as the independent variable and mean amplitude as the dependent variable, a repeated ANOVA was performed. The results indicated that for the N1 component (170–260 ms), the main effect of stimulus type at the PO7 electrode was significant, *F*(2, 76) = 3.281, *p* = 0.043, η^2^_p_ = 0.079. Post hoc tests showed that the amplitude for happy stimuli was significantly greater than for sad stimuli, M_diff_ = 0.248, *p* = 0.047, and the amplitude for neutral stimuli was significantly greater than for sad stimuli, M_diff_ = 0.207, *p* = 0.039.

With stimulus type as the independent variable and negative peak amplitude as the dependent variable, a repeated ANOVA was performed. The results indicated that the main effect of stimulus type at the PO7 electrode was significant, *F*(2, 76) = 3.799, *p* = 0.027, η^2^_p_ = 0.091. Post hoc tests showed that the amplitude for happy stimuli was significantly greater than for sad stimuli, M_diff_ = 0.698, *p* = 0.023, and the amplitude for neutral stimuli was significantly greater than for sad stimuli, M_diff_ = 0.585, *p* = 0.009.

**Figure 5:**
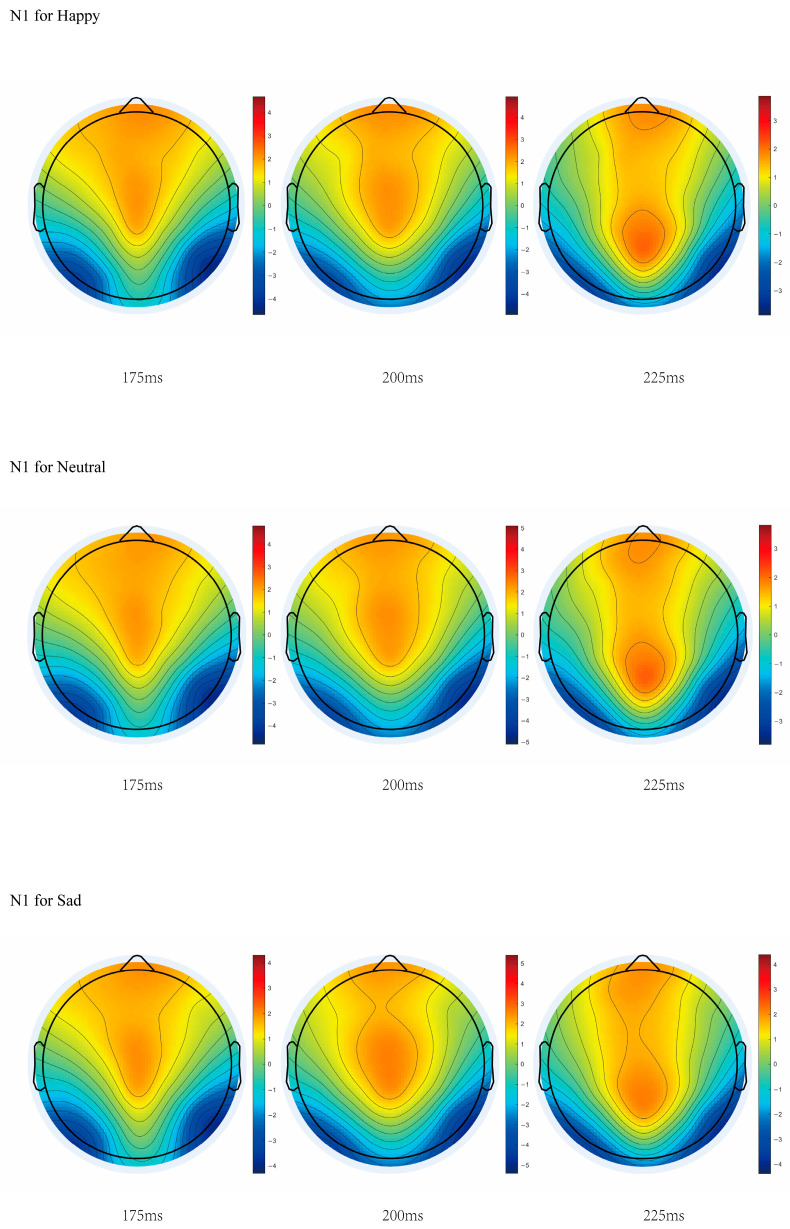
Topographic map of the N1 component.

On the target electrodes, there were no significant differences in peak latency. The details are shown in Figure 5.

#### 3.2.3. Analysis of P2p Component

With stimulus type as the independent variable and mean amplitude as the dependent variable, a repeated ANOVA was performed. The results indicated that the main effect of stimulus type at the PO7 electrode was significant, *F*(2, 76) = 28.190, *p* < 0.001, η^2^_p_ = 0.426. Post hoc comparisons showed that the amplitude for neutral stimuli was significantly greater than for happy stimuli, M_diff_ = 0.327, *p* = 0.002, significantly greater than for sad stimuli, M_diff_ = 0.992, *p* < 0.001, and the amplitude for happy stimuli was significantly greater than for sad stimuli, M_diff_ = 0.664, *p* < 0.001. At the PO8 electrode, the main effect of stimulus type was significant, *F*(2, 76) = 14.140, *p* < 0.001, η^2^_p_ = 0.271. Post hoc tests showed that the amplitude for neutral stimuli was marginally significantly greater than for happy stimuli, M_diff_ = 0.193, *p* = 0.056, significantly greater than for sad stimuli, M_diff_ = 0.662, *p* < 0.001, and the amplitude for happy stimuli was significantly greater than for sad stimuli, M_diff_ = 0.469, *p* = 0.001. At the Oz electrode, the main effect was significant, *F*(2, 76) = 14.499, *p* < 0.001, η^2^_p_ = 0.276. Post hoc tests showed that the amplitude for neutral stimuli was significantly greater than for sad stimuli, M_diff_ = 0.628, *p* < 0.001, and the amplitude for happy stimuli was significantly greater than for sad stimuli, M_diff_ = 0.464, *p* < 0.001.

With stimulus type as the independent variable and peak amplitude as the dependent variable, a repeated ANOVA was performed. The results indicated that the main effect of stimulus type at electrode PO7 was significant, *F*(2, 76) = 21.606, *p* < 0.001, η^2^_p_ = 0.362. Post hoc comparisons indicated that neutral stimuli elicited significantly larger amplitudes than happy, M_diff_ = 0.384, *p* = 0.001, and sad stimuli, M_diff_ = 1.206, *p* < 0.001, and happy stimuli showed significantly larger amplitudes than sad stimuli, M_diff_ = 0.822, *p* < 0.001. Similarly, a significant main effect was observed at PO8, *F*(2, 76) = 11.978, *p* < 0.001, η^2^_p_ = 0.238, with neutral stimuli evoking larger amplitudes than both happy, M_diff_ = 0.279, *p* = 0.035, and sad stimuli, M_diff_ = 0.854, *p* < 0.001, and happy stimuli again exceeding sad stimuli, M_diff_ = 0.575, *p* = 0.005. At electrode Oz, the main effect was also significant, *F*(2, 76) = 11.158, *p* < 0.001, η^2^_p_ = 0.227. Post hoc comparisons indicated that neutral stimuli marginally exceeded happy stimuli, M_diff_ = 0.263, *p* = 0.058, and were significantly larger than sad stimuli, M_diff_ = 0.714, *p* < 0.001, while happy stimuli remained significantly larger than sad stimuli, M_diff_ = 0.451, *p* = 0.010.

**Figure 6:**
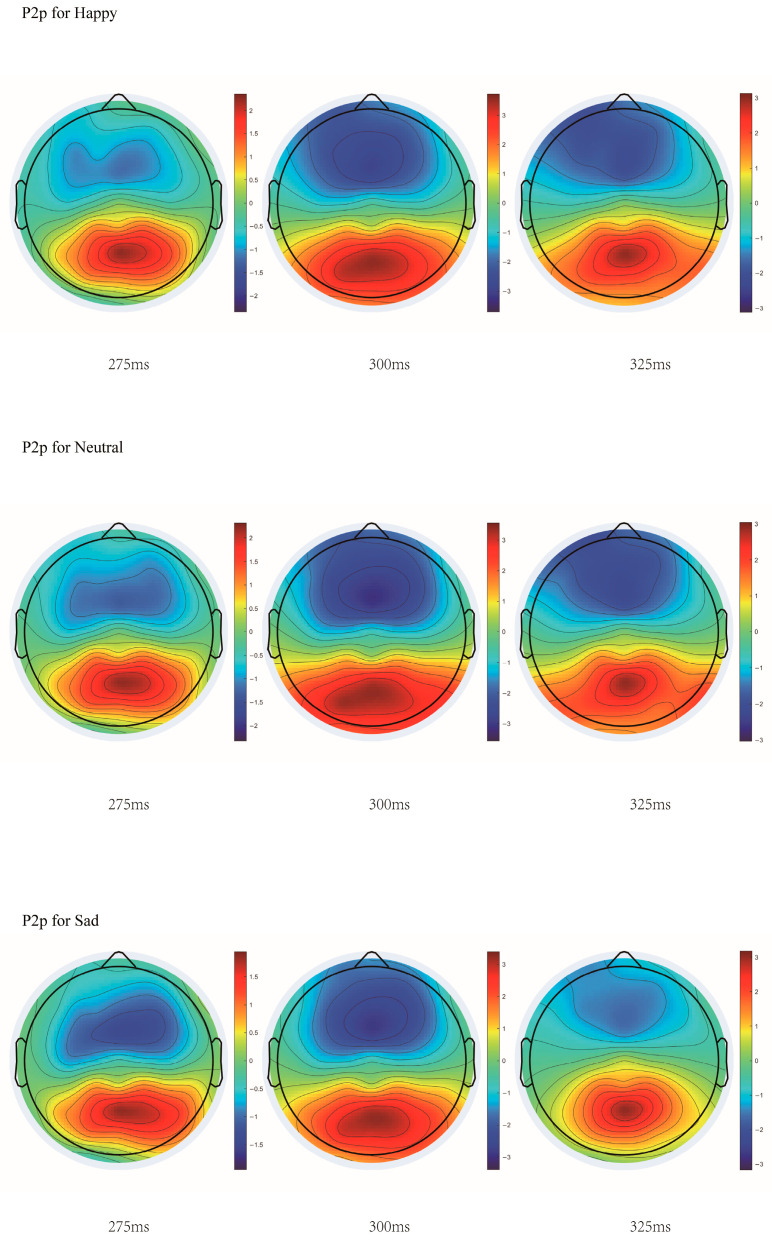
Topographic map of the P2p component.

Analysis of latency yielded a significant main effect of stimulus type only at Oz, *F*(2, 76) = 3.461, *p* = 0.036, η^2^_p_ = 0.083, with happy stimuli eliciting longer latencies than sad stimuli, M_diff_ = 7.011, *p* = 0.032. No other pairwise comparisons reached significance. The details are shown in Figure 6.

### 3.3. Linear Mixed Model

To assess the relationship between electrophysiological signals and behavior, we fitted a linear mixed-effects model (pse_value ~ amplitude × electrode × stimulitype + (1|ID)).

Regarding the C1 component, the main effect of amplitude was non-significant, *F*(1, 110.94) = 1.63, *p* = 0.205). However, its interaction with stimulus type showed a marginal trend, *F*(2, 296.26) = 2.34, *p* = 0.098). Neither the main effect of electrode site (PO7, PO8, OZ) nor any of its interactions were significant (all *p*s > 0.92), indicating consistent effects across these locations. Simple slope analysis (emmeans::emtrends, averaged across electrodes) revealed a significant negative relationship between C1 amplitude and PSE specifically for sad stimuli (b = −0.060, SE = 0.025, 95% CI [−0.109, −0.011]), where larger amplitudes were associated with lower PSE values. This relationship was non-significant and near zero for both happy (b = −0.014, 95% CI [−0.069, 0.040]) and neutral (b = 0.002, 95% CI [−0.048, 0.053]) conditions. Pairwise comparisons of the slopes (Tukey-corrected) showed that the negative slope for sad stimuli differed marginally from that for neutral stimuli (difference = 0.062, *p* = 0.094), but not from the happy condition (*p* = 0.307). These results suggest that the influence of C1 amplitude on perceptual thresholds is most pronounced for sad faces, implying that early visual processing may preferentially enhance sensitivity to negative information.

For the N1 component, the main effect of amplitude showed a marginal, overall negative trend (*t* = −1.83, *p* = 0.068). Critically, a significant amplitude-by-emotion interaction was observed, *F*(2, 296) = 4.17, *p* = 0.016. Simple slope analysis indicated a significant negative correlation between amplitude and PSE exclusively in the happy condition (b = −0.033, 95% CI [−0.057, −0.008], *p* = 0.040. The slope for happy stimuli was significantly different from those for both neutral (*p* = 0.042) and sad (*p* = 0.026) stimuli. Effects were again consistent across electrode sites (PO7, PO8, OZ), as neither the main effect nor any interactions involving the electrode were significant (all *p*s > 0.70).

Analysis of the P2p component revealed a non-significant main effect of amplitude, *F*(1, 109.52) = 0.03, *p* = 0.856), and a marginal amplitude-by-emotion interaction, *F*(2, 297.25) = 2.37, *p* = 0.095. Electrode site and its interactions were non-significant (all *p*s > 0.92), confirming effect consistency. Simple slope analysis (averaged across electrodes) showed only a weak, non-significant negative trend between P2p amplitude and PSE in the happy condition (b = −0.018, SE = 0.016, 95% CI [−0.049, 0.013]). The relationships for neutral (b = 0.003, 95% CI [−0.027, 0.033]) and sad (b = 0.022, 95% CI [−0.010, 0.053]) conditions were also non-significant and near zero. Pairwise slope comparisons yielded a marginal trend for the happy condition slope to differ from the sad condition slope (difference = −0.040, *p* = 0.078), but this was not statistically significant (other *p*s > 0.46). Overall, P2p amplitude exhibited a notably weaker predictive relationship with perceptual thresholds compared to the earlier C1 and N1 components, with only a slight negative trend observed in the happy context.

## 4. Discussion

We examined whether stimulus reward properties systematically modulate numerical perception and explored the underlying neural correlates. Happy, neutral, and sad facial expressions were employed as rewarding, neutral, and punishing stimuli, respectively, to assess how social reward value influences number sense, with simultaneous EEG recording. Behavioral responses were evaluated using the point of subjective equality (He et al., 2015), while EEG activity was analyzed from numerosity-sensitive electrodes PO7, PO8, and Oz (Park et al., 2015; Yao et al., 2023). The following sections discuss the main findings separately.

### 4.1. Moderation of Social Rewarding/Punishing Stimuli

Behavioral results revealed that, compared to neutral and punishing stimuli, social reward stimuli elicited a significantly higher point of subjective equality (PSE) in numerosity judgments, indicating a systematic underestimation of reward-related quantities. In contrast, punishing stimuli yielded a significantly lower PSE relative to neutral stimuli, reflecting an overestimation effect. These findings are consistent with earlier reports of emotional influences on numerical perception (Lewis et al., 2018). A potential explanation lies in value-driven attentional salience mechanisms: reward-associated stimuli automatically capture attention (Anderson et al., 2011), and attentional allocation enhances perceptual salience (Carrasco et al., 2004). Specifically, reward-driven attention increases the perceived contrast of stimuli relative to neutral items (Qin et al., 2021). Previous work has demonstrated that enhanced physical contrast under mixed-stimulus conditions leads to numerical underestimation (Lei & Reeves, 2018, 2023). The present results suggest that attentionally mediated contrast enhancement may override baseline perceptual constraints, implying that reward-induced numerical underestimation originates from contrast modulations driven by attentional capture. Notably, while previous studies have emphasized reward’s influence on behavioral performance, this study is the first to demonstrate that intrinsic reward properties of stimuli can directly modulate the number sense process, resulting in underestimation for rewards and overestimation for punishments.

### 4.2. Early Moderation of Rewarding Stimuli on Number Sense

EEG results for the C1 component (50–100 ms post-stimulus) revealed a significant main effect of stimulus type at electrodes PO7, PO8, and Oz. Post hoc tests indicated that across all three sites, happy stimuli elicited significantly smaller amplitudes than sad stimuli, and neutral stimuli also elicited significantly smaller amplitudes than sad stimuli. No other pairwise comparisons reached significance.

The C1 component, originating in the primary visual cortex, and the electrodes PO7, PO8, and Oz—established as key sites for early numerical perception (Park et al., 2015)—collectively indicate that reward-related properties modulate number sense at an initial, likely unconscious stage of visual processing. As the C1 manifests as a negative deflection at these electrodes, the larger absolute amplitudes evoked by happy and neutral stimuli relative to sad stimuli suggest that rewarding and neutral conditions elicit stronger early neural activity in the visual cortex compared to punishing stimuli. However, no significant difference was observed between happy and neutral stimuli. These findings align with previous reports that reward anticipation enhances EEG responses in early time windows (Verma, 2024). It further supported the role of reward valuation in shaping early sensory processing.

### 4.3. Sensitivity of N1 to Punishing Stimuli

For the N1 component (170–260 ms post-stimulus), a significant main effect of stimulus type was observed at electrode PO7. Post hoc analyses revealed that happy stimuli elicited a significantly larger amplitude than sad stimuli, and neutral stimuli also elicited a significantly larger amplitude than sad stimuli. Given that the N1 manifests as a negative deflection, these results indicate that sad stimuli actually evoked a stronger neural response compared to both happy and neutral stimuli. Thus, relative to rewarding and neutral conditions, punishing stimuli enhanced EEG activity in the N1 time window. This amplification of N1 amplitude by negative stimuli is consistent with prior findings (Sun et al., 2012).

The N1 component, which is closely linked to number sense (Yao et al., 2023), demonstrates high sensitivity to quantitative variation and reflects the rapid, direct extraction of numerosity within the human visual pathway (Park et al., 2015; Van Rinsveld et al., 2020). The enhanced N1 amplitude elicited by punishing stimuli at the PO7 electrode suggests that punishment-related properties exert a modulatory influence on numerical processing. From a temporal perspective, these findings indicate that reward-related attributes are processed earlier than punishment-related attributes during visual number perception.

### 4.4. Inhibitory Effect on Rewarding Stimuli in P2p

For the P2p component (270–360 ms post-stimulus onset), neutral stimuli elicited significantly larger amplitudes than both rewarding and punishing stimuli across electrodes PO7, PO8, and Oz. The P2p component, localized in parietal regions, is known to reflect sensitivity to numerical changes (Fornaciai et al., 2017), and is thought to represent relatively pure quantity extraction rather than integration with non-numerical visual attributes (Grasso et al., 2022). The enhanced amplitude for neutral stimuli suggests that, during the P2p time window, the influence of reward and punishment properties is suppressed, thereby helping to preserve the accuracy of numerosity processing. This pattern further supports the view that the P2p reflects a perceptual stage dedicated to the extraction of abstract numerical information. This may reflect that the brain exerts suppressive control over task-irrelevant reward attributes to ensure the accuracy of numerical information retrieval. The pattern of adopting distinct processing strategies across different temporal windows demonstrates a neural mechanism of dynamic regulation. This bears resemblance to the encoding dynamics in arithmetic rule processing: the hippocampus tends to employ a temporally stable ‘static coding’ to maintain rule information, whereas the parahippocampal cortex exhibits rapidly shifting dynamic coding (Kutter et al., 2022). While the tasks differ, this reflects that the brain adopts flexible strategies when processing mathematically related tasks.

It is worth noting that although the findings of this study highlight biases induced by reward attributes, the suppression of this bias by the P2p component shares similarities with the characteristic of transitive inference, which transcends the influence of associative values and reward reinforcement and relies primarily on implied order relationships (Gazes et al., 2012; Jensen et al., 2017). This similarity thus raises a question: Are non-symbolic number sense and transitive inference related? This warrants further exploration.

### 4.5. Limitations and Future Directions

This study sought to investigate the influence of stimulus reward properties on numerical perception; however, several limitations should be noted.

Firstly, although reward type was manipulated, the effect of reward magnitude—such as the differential impact of small versus large rewards on number sense—was not explored, leaving open questions regarding dose–response relationships in reward-based modulation.

Secondly, even though the stimuli we employed can convey emotions such as happiness and sadness (Jaeger et al., 2019; Jaeger & Ares, 2017), in real-world emoji usage, individuals may still hold varying interpretations of their meanings. In future research, we plan to utilize emojis with ambiguous or contradictory meanings, such as the classic smiling emoji or the laughing-with-tears emoji. Participants will be differentiated through pre-experiment surveys—grouping those who perceive the smiling emoji as positive in one group and those who interpret it as negative in another—to examine the transferability of stimulus attributes. Certainly, we also verbally inquired with participants after the experiment about their interpretations of these facial expressions. The vast majority of participants indicated that these expressions indeed corresponded to positive, negative, and neutral emotions, which aligns with our experimental hypotheses.

Furthermore, primary visual attributes were not systematically manipulated but were instead assigned randomly. Although, the analysis of JND indicated that perceptual sensitivity to numerical differences did not differ significantly across the happy, neutral, and sad conditions.

Finally, as the sample consisted exclusively of young adults, the results should not be broadly generalized to other age groups without further validation in more diverse populations.

Although positive emotions are often treated as social rewards, the reward and emotional systems are inherently dissociable (Chakravarthula & Padmala, 2023). The present study did not fully disentangle these constructs. Future work should therefore include control conditions that directly contrast monetary with emotional rewards to isolate their respective contributions. Also, future research might assess whether non-social rewards (e.g., monetary or food incentives) similarly modulate number sense, thereby extending the generalizability of these findings beyond social contexts. Additionally, it is worthwhile to adopt network-level analyses (e.g., reward–attention or frontoparietal systems), which would further elucidate how reward attributes engage distributed neural circuitry during numerical processing, thus strengthening the methodological rigor and the validity of the conclusions.

## 5. Conclusions

The present study demonstrates that reward properties of stimuli induce underestimation in number sense, whereas punishment properties lead to overestimation, reflecting a top-down modulatory influence of higher cognitive processes on numerical perception. EEG findings further reveal that this modulation occurs as early as the primary visual cortex, with reward-related effects emerging prior to those associated with punishment. These results underscore the potent and fundamental nature of cognitive influence on number sense, indicating that numerical processing is automatically adjusted by reward relevance even before explicit decision-making begins. Concurrently, the suppression of stimulus reward effects during the P2p component suggests that this process is subject to dynamic regulation. These findings challenge the conventional view of number sense as a relatively autonomous perceptual process, supporting instead the notion that human quantity discrimination is not a pure reflection of numerical magnitude but is systematically shaped by task-irrelevant motivational factors.

## Figures and Tables

**Table 1:** Descriptive statistics of Experiment 1.

Stimuli Type	RT (*M* ± *SD*) (Second)	PSE (*M* ± *SD*)
Happy	0.525 ± 0.200	12.700 ± 0.436
Sad	0.521 ± 0.199	11.352 ± 0.615
Neutral	0.538 ± 0.210	12.038 ± 0.224

## Data Availability

The data presented in this study are openly available in [OSF] at https://osf.io/2xm57/files/osfstorage, accessed on 25 February 2026.
